# Relations of the Vaginal Secretions to Puerperal Fever

**Published:** 1893-11

**Authors:** 


RELATION OF THE VAGINAL SECRETIONS TO PUERPERAL FEVER.—(Zeitschr. f. Geburts-Nulfe.) Doderlein examined the secretions in one hundred and ninety-five cases of pregnant women with reference to the presence of pathogenic organisms. In normal secretions only fission fungi were found while bacilli and cocci occurred in great number and variety in those of disease. The percentage of healthy cases was 55.4 per cent., of pathological 44.6 per cent. In primiparæ the proportion of normal cases was a little larger, amounting to about 64 per cent. The author suggests the litmus reaction as a ready means of distinguishing healthy from morbid conditions of the vaginal secretions. Normally the reaction of the vaginal mucus is strongly acid, while in disease it is faintly acid, neutral or alkaline. Experiments show that the acid condition of the vaginal secretions is produced by the innoxious organisms which have their habit in the healthy vagina. The author found by culture experiments that these organisms when present in great abundance, were inimical to the growth of the staphylococcus. A similar observation has been made by Bumm.

The author's conclusions for the practical obstetrician are to the effect that in women whose vaginal secretions present the normal litmus reaction, a feverless childbed is assured, provided they escape infection from without.

In morbid conditions of the vaginal secretions pathogenic organisms are liable to be transported into the uterus on the examining finger. Without internal manipulation of any kind during labor the danger of infection is almost nil.

In private practice it is generally impracticable to distinguish between healthy and morbid vaginal secretions. The prophylaxis for every case must be such as to protect against all possible sources of infection.

In hospital clinics the litmus reaction serves to separate the diseased from the normal cases.

Antiseptic irritation of the vagina for disinfection is not reliable. The irrigation fluid does not penetrate all the folds and recesses of the passages. Steffeck and Doderlein have practiced irrigation combined with thorough friction with the fingers, and lubrication is re-supplied by rubbing the vaginal surfaces with mollin.

For diseased cases they recommend the use of the disinfectant douche during the latter weeks of pregnancy as more satisfactory than attempts at sterilizing the passages during labor. A one per cent solution of lactic acid answers the purpose. The secretions gradually take on a healthy character. The douching is discontinued when the litmus test gives a constant acid reaction.—*Brooklyn Med. Jour.*

